# The role of Hippo pathway in ferroptosis

**DOI:** 10.3389/fonc.2022.1107505

**Published:** 2023-01-11

**Authors:** Jiangxia Xiang, Mengmeng Jiang, Xing Du

**Affiliations:** ^1^ Department of Traumatology, Chongqing Emergency Medical Center, Chongqing University Central Hospital, Chongqing, China; ^2^ Department of Medical Oncology, The Third Central Hospital of Tianjin, Tianjin, China; ^3^ Department of Orthopedics, The First Affiliated Hospital of Chongqing Medical University, Chongqing, China; ^4^ Orthopedic Laboratory of Chongqing Medical University, Chongqing, China

**Keywords:** Hippo pathway, YAP, ferroptosis, iron metabolism, ROS

## Abstract

The role of Hippo pathway in ferroptosis

The Hippo pathway is mainly composed of mammalian serine/threonine (Ste20)like kinases 1/2 (MST1/2), large tumor suppressor 1/2 (LATS1/2), and transcriptional coactivator Yes-associated protein (YAP), and is closely related to cell growth, survival, proliferation, and migration; tissue and organ size control; and tumorigenesis and development. Ferroptosis is a regulated form of cell death characterized by the accumulation of iron-dependent reactive oxygen species (ROS) and the depletion of plasma membrane polyunsaturated fatty acids (PUFAs), which is caused by the imbalance of oxidation and the antioxidant system. This article elaborates the role of Hippo pathway in ferroptosis, providing ideas for the regulation of cell fate and the treatment of tumors.

## Introduction

The Hippo pathway can accept and integrate upstream signals and guide cell gene transcription and biological behavior through the transcriptional coactivator Yes-associated protein (YAP), which is closely related to cell growth, survival, proliferation, and migration; tissue and organ size control; and tumor occurrence and development. Ferroptosis is a regulated form of cell death characterized by the accumulation of iron-dependent reactive oxygen species (ROS) and the depletion of plasma membrane polyunsaturated fatty acids (PUFAs), which is caused by the imbalance of oxidation and the antioxidant system *in vivo*. In this review, the role of Hippo pathway in ferroptosis was reviewed and discussed.

## Hippo pathway and Yes-associated protein

### The core molecule of Hippo pathway

**Figure 1:**
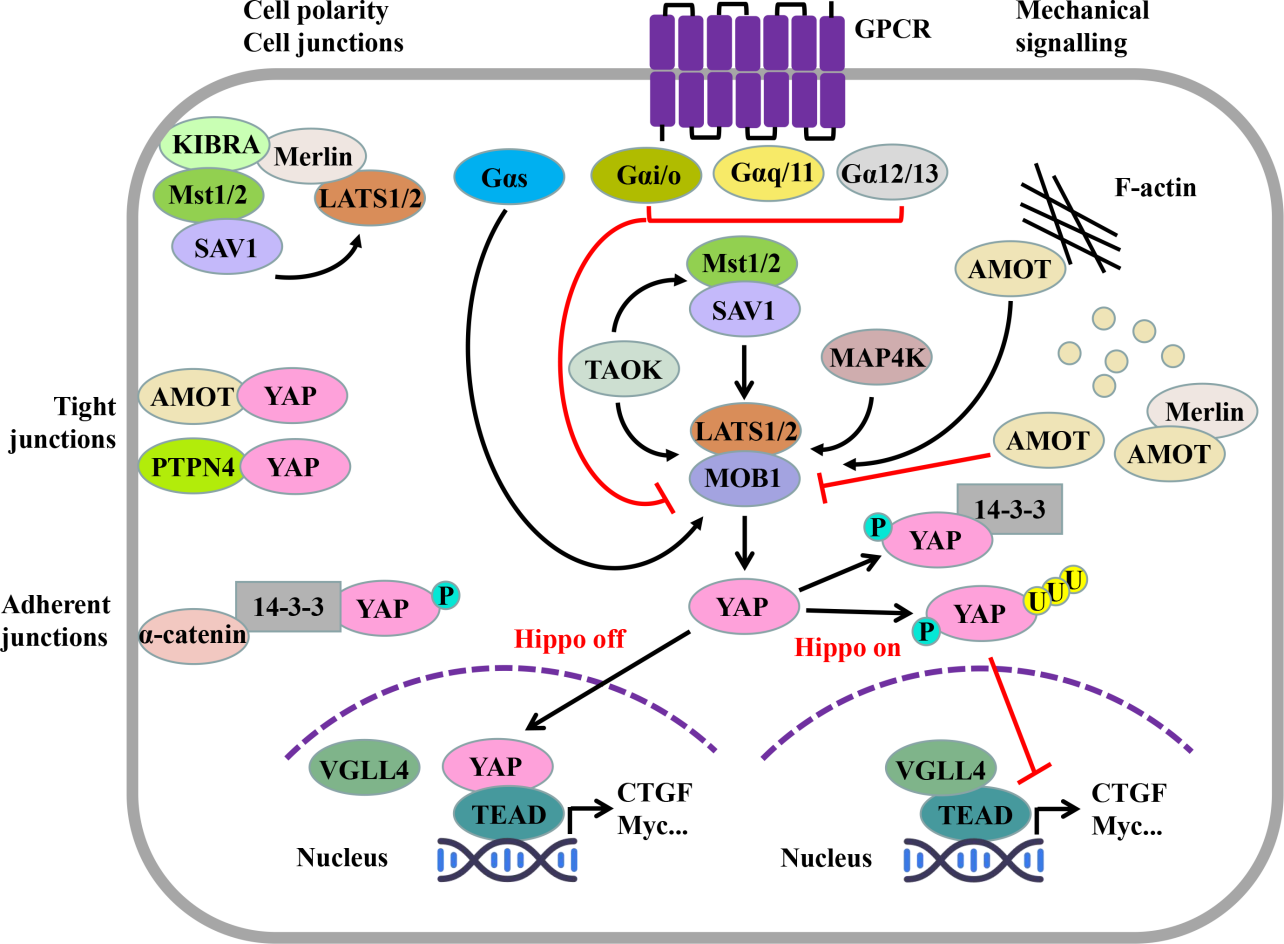
Hippo pathway and its regulatory factors. The Hippo pathway consists of serine/threonine kinases MST1/2 and LATS1/2, regulatory molecules SAV1 and MOB1, and effector molecules YAP. When the Hippo pathway is not activated, YAP, as a transcriptional coactivator, can enter the nucleus and combine with TEAD competitively, thus inducing the transcription of target genes. When the Hippo pathway is activated, activated MST1/2 can phosphorylate LATS1/2, and then, YAP is further phosphorylated. Phosphorylated YAP can be captured by 14-3-3 proteins in the cytoplasm or degraded through the SCF^β-TRCP^ E3 ligase-mediated ubiquitin proteasome pathway. The Hippo pathway is regulated by several upstream signals. Cell connectivity and cell polarity components, such as KIBRA, AMOT, PTPN4, and α-catenin, are a classical upstream regulator of the Hippo pathway; the effect of the GPCR on the Hippo pathway depends on its α subunit type; and mechanical cues can regulate the Hippo pathway by affecting LATS1/2 activity through the cytoskeleton. (MST1/2, mammalian Ste20-like kinase 1/2; SAV1, Salvador family WW domain-containing protein 1; LATS1/2, large tumor suppressor factor 1/2; MOB1, MOB kinase activating factor 1; YAP, Yes-associated protein; TAOK, TAO kinase; MAP4K, mitogen-activated protein kinase kinase kinase kinase; TEAD, TEA domain transcription factor family; VGLL4, vascular-like protein 4; KIBRA, kidney brain protein; Merlin, NF2, neurofibroma protein 2; AMOT, angiomotin; PTPN14, protein tyrosine phosphatase non-receptor type 14; AJ, adherent junction; TJ, tight junction; GPCR, G protein–coupled receptor.).

The Hippo pathway is mainly composed of mammalian Ste20-like kinases 1/2 (MST1/2), large tumor suppressor 1/2 (LATS1/2), regulator family Salvador family WW domain-containing protein 1 (SAV1), Mps One Binder (MOB) kinase activator 1 (MOB1), and transcriptional coactivator YAP (1–4) (**Figure 1**).

After MST1/2 is activated by the upstream kinase thousand-and-one amino acid kinase (TAOK) (5) or dimerization itself (6), it can combine with SAV1 through its C-terminal Sav/RASSF/Hpo (SARAH) domain to phosphorylate SAV1 (2). Phosphorylated SAV1 can interact with LATS1/2 to assist LATS1/2 phosphorylation (7). At the same time, the regulatory protein MOB1 of LATS1/2 is phosphorylated by MST1/2 (T12, T35) (8). Phosphorylated SAV1 and MOB1 assist MST1/2 to phosphorylate the hydrophobic motif (HM) outside the active center of LATS1/2 kinase (T1041, T1079) (9, 10). After HM is phosphorylated, with the help of MOB1, LATS1/2 finally undergoes self-phosphorylation (S87, S909), thus being fully activated (4, 8) (**Figure 1**).

TAOK can phosphorylate MST1 (T183) and MST2 (T180) or directly phosphorylate LATS1/2 (4, 5) (**Figure 1**). In addition to TAOK and MST1/2, MAP4K4/6/7 and MAPK4K1/2/3 in the MAP4K (mitogen-activated protein kinase kinase kinase kinase) family can also directly activate LATS1/2 (11) (**Figure 1**). The direct activation of MAP4K on LATS1/2 is independent of MST1/2, and when MAP4K and MST1/2 genes are knocked out, YAP phosphorylation is still in progress; thus, there may be kinases other than MAP4K and MST1/2, such as TAOK, that phosphorylate YAP through LATS1/2 (11, 12).

LATS1/2 activated by phosphorylation can recognize the five HXRXXS motifs of YAP (13) and phosphorylate them (S61, S109, S127, S164, and S381) (14). Among them, after the phosphorylation of serine (S127) at position 127 of YAP, 14-3-3 proteins in the cytoplasm can capture it and locate it in the cytoplasm (13) (**Figure 1**). The phosphorylation of serine (S381) at position 381 can induce creatine kinase CK1 to phosphorylate serine (S384) at position 384 of YAP; thus, recruiting Skp1-Cullin-F-box (SCF) ^β-TRCP^ E3 ubiquitin ligase degrades YAP through the ubiquitin proteasome pathway (14) (**Figure 1**).

### Effective molecule of the Hippo pathway—Yes-associated protein

When the Hippo pathway is not activated, YAP is located in the nucleus; interacts with the transcription factors Transcriptional enhanced associate (TEA) domain (TEAD) 1–4; starts the expression of genes such as the connective tissue growth factor (CTGF), cysteine-rich angelic indicator 61 (Cyr61), myelocytosis oncogene (Myc), and survivin; and promotes cell growth and proliferation (15) (**Figure 1**). When the Hippo pathway is activated, YAP is phosphorylated by LATS1/2, or located in the cytoplasm by binding with 14-3-3 proteins (13), or degraded by the proteasome *via* SCF^β-TRCP^ E3 ubiquitin ligase (14). At this time, vascular-like protein 4 (VGLL4) inhibits its transcriptional activity by combining its TONDU (TDU) domain with the transcription factor TEAD (16, 17) (**Figure 1**).

The nuclear entry and exit behavior of TEAD, as a transcription factor downstream of the Hippo pathway, is not regulated by MST1/2, LATS1/2, MAP4K, and other Hippo pathway–related molecules. It is known that the p38 MAPK signaling pathway plays an important role in regulating the nuclear entry and exit behavior of TEAD. Under the stimulation of continuous high osmotic pressure, p38 was directly combined with TEAD to make TEAD nucleate (18). In addition, the p38 MAPK signal pathway can promote the polymerization of F-actin, inhibit Hippo pathway, and promote YAP to enter the nucleus (19).

In addition to TEAD1–4, YAP can interact with Runx1/2, BRD4, Smad, and other transcription factors after entering the nucleus (2, 10, 20).

### Regulation mode of Hippo pathway

The Hippo pathway is mainly regulated by mechanical signal, cell polarity and cell connection, and G protein–coupled receptor (GPCR) (2, 4, 21, 22) (**Figure 1**).

F-actin is one of the important regulatory factors of Hippo pathway. The presence of F-actin inhibitors, such as bravastatin, the fillin/actin depolymerization factor (cofilin), gelsolin, and β-coactin/capping protein Z (CapZ), leads to a decrease in the transcriptional activity of YAP-related genes, while the induction of F-actin polymerization enhances the activity of YAP (23, 24), and F-actin regulates YAP activity in a manner that is LATS1/2-dependent rather than MST1/2-dependent (21).

The proteins involved in the formation of cell polarity and cell connection can also regulate the Hippo pathway. On the one hand, kidney and brain (KIBRA) protein on the top skin layer of cells can recruit LATS1/2 to the cell membrane through Merlin (also known as neurofibromin 2, NF2). On the other hand, it can recruit MST1/2 to the cell membrane through SAV1, thus mediating the phosphorylation of LATS1/2 by MST1/2 (25) and inhibiting the activity of YAP (**Figure 1**). In this process, MST1/2 is not phosphorylated (26).

Angiomotin (AMOT), which is related to cell polarity formation, can bind to F-actin through its N-terminal (27). When F-actin is destroyed, AMOT is phosphorylated by LATS1/2 and separated from F-actin (28). Then, AMOT, as a scaffold protein, binds to MST1/2, LATS1/2, and YAP, respectively, and mediates the phosphorylation of MST1/2 to LATS1/2 and LATS1/2 to YAP (9) (**Figure 1**). In addition, AMOT can bind to Merlin, expose the domain that Merlin binds to LATS1/2, and make LATS1/2 bind to it and be activated, thereby inhibiting the activity of YAP (29) (**Figure 1**).

AMOT and protein tyrosine phosphate non-receptor type 14 (PTPN14), the cell tight junction–related protein, can bind to the WW domain of YAP through the PPXY motif, making them locate at the cell tight junction (30). When cells are in high density, α-catenin can combine 14-3-3 proteins with phosphorylated YAP to promote its cytoplasmic localization (31) (**Figure 1**).

GPCR can be combined with a variety of hormones and growth factors and regulate the activity of YAP through LATS1/2 (**Figure 1**). In addition, different types of subunits α of G protein show different actions: Gαs can activate LATS1/2, phosphorylate YAP, and promote its cytoplasmic localization, while Gαi/o, Gαq/11, and Gα12/13 inhibit the activity of LATS1/2 and increase the transcriptional activity of YAP (32).

## Ferroptosis

Cell death is divided into regulated cell death (RCD) and accidental cell death (33). RCD refers to the form of cell death that is regulated by the cell’s own gene and depends on the signal pathway related to cell death, mainly including apoptosis, necrotic apoptosis, and pyroptosis. Accidental cell death refers to a form of death that does not depend on molecular signal pathways when physical or chemical factors cause damage to cells (34). RCD plays an important role in the normal development of organisms and the maintenance of homeostasis. In recent years, with the discovery of ferroptosis and the in-depth study of its mechanism, ferroptosis is also considered as a kind of controllable cell death (35, 36).

Ferroptosis is a regulated form of cell death characterized by the accumulation of iron-dependent lipid ROS and the depletion of plasma membrane PUFAs, which is caused by the imbalance of oxidation and the antioxidant mechanism *in vivo* (37, 38).

As a new and controllable form of cell death, ferroptosis first came into the view of scientists in 2003. STOCKWELL laboratory (39) found that erastin (eradicator of Ras and ST) and Ras-selective lethal 3 (RSL3), two oncogenes encoded by erastin protein and RSL protein, had a specific lethal effect on H-ras proto-oncogene (HRAS) mutant cells when they screened small molecules with a specific lethal effect on the small GTPase of the RAS family [HRAS, N-ras proto-oncogene (NRAS), and K-ras proto-oncogene (KRAS)] of mutant cells, and this lethal effect did not depend on BCL-2, BAX, BAK, and other apoptosis-related proteins, It is also different from the known forms of non-programmed cell death such as cell necrosis (38). Subsequent studies have proven that cell death caused by erastin and RSL3 is closely related to the accumulation of iron-dependent lipid ROS (38). This form of cell death is named ferroptosis.

### Mechanism of ferroptosis

#### Production of reactive oxygen species

Molecules containing oxygen atoms that have not been completely reduced are called ROS. The active oxygen in organisms mainly includes superoxide (O_2_•^-^), peroxide (H_2_O_2_ and ROOH), and oxygen free radicals (HO• and RO•) (40, 41).

Intracellular ROS are mainly produced by the electron transfer chain (42, 43) and Nicotinamide Adenine Dinucleotide Phosphate (NADPH) oxidase (NOX) in the energy metabolism of mitochondria (44) and the biological reactions catalyzed by oxidase, such as the formation of uric acid from hypoxanthine and the oxidation of fatty acids by peroxisomes (43). In addition, radiation and air pollutants can induce cells to produce living oxygen (42), and neutrophils and macrophages can produce living oxygen when they are activated to exercise anti-infection function (45).

**Figure 2:**
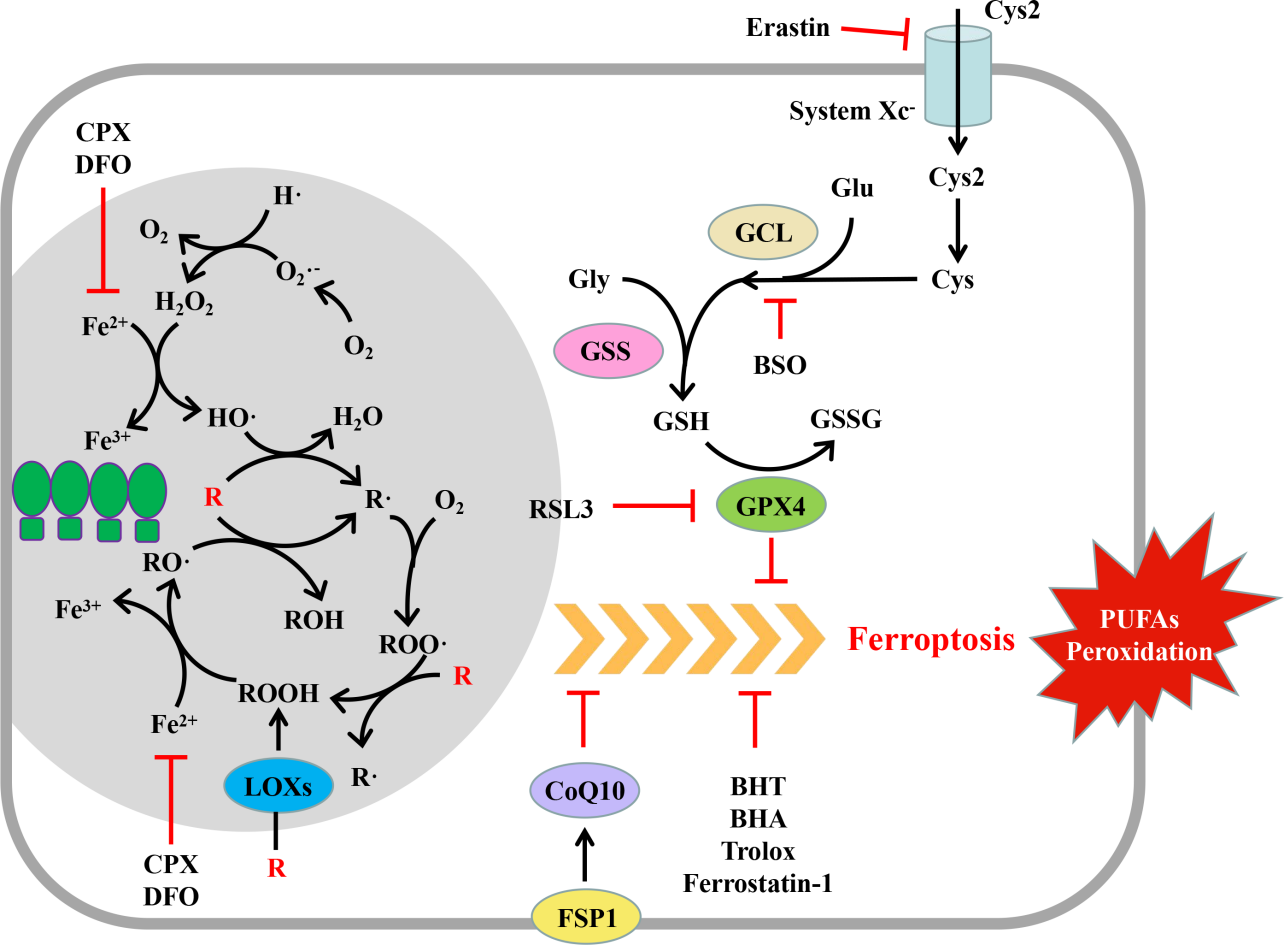
Mechanism and regulation of ferroptosis. Ferroptosis is a regulated form of cell death characterized by the peroxidation of polyunsaturated fatty acids (PUFAs) of the plasma membrane. Peroxide PUFAs are mainly produced in the Fe^2+^-dependent chain reaction mediated by reactive oxygen species (ROS) and lipoxygenases (LOXs). GPX4 and FSP1 can eliminate excessive PUFAs while producing them, thus maintaining the homeostasis of the intracellular environment. When the cell oxidation and antioxidant systems are out of balance, the PUFAs of the plasma membrane are oxidized in large quantities, which changes the permeability of the plasma membrane, leading to cell death. (R, PUFAs; PUFAs, polyunsaturated fatty acids; GPX, glutathione peroxidase; FSP1, ferroptosis inhibitor protein 1; CoQ10, coenzyme Q10; Cys2, cystine; Cys, cysteine; Glu, glutamic acid; Gly, glycine; GCL, glutamate cysteine ligase; GSS, glutathione synthetase; GSH, reduced glutathione; GSSG, oxidized glutathione; erastin, ferroptosis inducer; RSL3, ferroptosis inducer; BSO, buthionine-(S,R)-maple imine; BHT, butylated hydroxytoluene; BHA, tert butyl hydroxyanisole; DFO, deferoxamine; CPX, ciclopidone).

In the said process, O_2_ is oxidized to O_2_•^-^ by the corresponding oxidase, and then, O2•^-^ can transfer an electron to Fe^3+^, or the disproportionation reaction occurs under the action of superoxide dismutase to generate H_2_O_2_ and O_2_. H_2_O_2_ can accept one electron of Fe^2+^ to form HO• and HO^-^ (42, 46) (**Figure 2**).

Lipid ROS can be produced by a non-enzymatic lipid spontaneous oxidation reaction or lipoxygenase (46–48) (**Figure 2**). Spontaneous lipid oxidation is usually initiated by ROS. Take HO• as an example; HO• can capture the H of PUFAs (R) on the cell membrane to generate R• and further react with oxygen to generate ROO•. ROO• can further capture the H of another PUFA on the cell membrane to generate ROOH and a new R•, and ROOH can accept an electron of Fe^2+^ to form RO• and HO^-^. RO• and the newly generated R• can start a new round of oxidation reaction, form a vicious circle, and cause a large-scale oxidation of lipid on the cell membrane (47, 48).

Lipid ROS can also be formed by enzyme catalysis. Under the catalysis of lipoxygenase, PUFAs on the cell membrane can directly react with O_2_ to generate ROOH, which receives an electron of Fe^2+^ to form RO• and HO^-^, and then RO• starts the chain oxidation reaction of PUFAs on the cell membrane (43, 46).

In the said process, iron ions play a significant role as electron transmitters (48). Under normal conditions, the concentration of iron ions that can participate in the redox reaction is stable and maintained at 0.2–0.5 μMol/L (49); the remaining iron ions are stored in proteins such as heme and ferritin and do not participate in the redox reaction to avoid excessive production of ROS. However, under excessive oxidative stress, a high concentration of superoxide can release Fe^2+^ in iron-containing protein, thus promoting the generation of ROS (43, 48).

#### Antioxidant system

Under physiological conditions, excessive ROS produced in the body can be eliminated by a powerful antioxidant system. The antioxidant system is mainly composed of macromolecular enzymes such as superoxide dismutase, catalase, glutathione peroxidase, ascorbic acid peroxidase, and other small antioxidants such as vitamin C/E, carotenoids, and flavonoids (42, 50).

Superoxide dismutase (SOD) can catalyze the disproportionation reaction of O_2_•^-^ so that one molecule of O_2_•^-^ is oxidized to O_2_, and the other molecule is reduced to H_2_O_2_. There are three kinds of SOD isoenzymes in mammalian cells, namely, Cu/Zn SOD (the active center contains Cu/Zn ions), Mn SOD (the active center contains Mn^2+^), and catalase. Cu/Zn SOD is mainly distributed outside the cells and in the cytoplasm, and Mn SOD is mainly distributed in the mitochondria (51). Catalase mainly exists in peroxisomes, cytoplasm, and microsomes, which can catalyze H_2_O_2_ to decompose it into O_2_ and H_2_O and is one of the main forces to eliminate peroxides in the body (50).

Glutathione peroxidase (GPX) is the general name of a group of isoenzymes (GPX1–8). The active center of GPX contains selenocysteine residues, which can reduce H_2_O_2_/ROOH to H_2_O/ROH through reduced glutathione (GSH), and produce oxidized glutathione (GSSG) (52).

Mammalian cells contain four kinds of GPX, namely, GPX1–GPX4 isoenzymes: GPX1 is mainly found in the cytoplasm, nucleus, and mitochondria of the liver, lung, kidney tissue cells, and red blood cells. GPX2 mainly exists in the cytoplasm and nucleus of gastrointestinal cells. GPX3, known as plasma GPX3, is a secreted protein, which exists in the cytoplasm of different tissues and cells such as the kidney, lung, heart, and muscle. GPX4 widely exists in the cytoplasm, nucleus, and mitochondria of different tissues and cells and can combine with the membrane, thus, it is called phospholipid GPX (53).

GPX4 is the main regulatory molecule of ferroptosis (54). It can transform toxic lipid peroxide (ROOH) into non-toxic lipoid (ROH) through GSH so that the fluidity and integrity of the biofilm will not be damaged (43). Whether inhibiting the expression of GPX4 or its enzyme activity, or inhibiting the synthesis of GSH, it can induce the occurrence of ferroptosis (54, 55).

#### Oxidation and antioxidation imbalance

When the antioxidant system is inhibited or the active oxygen is hyperactive, the PUFAs on the plasma membrane are very sensitive to the active oxygen and lipoxygenase (48), and it is easy to generate ROOH. In the presence of Fe^2+^, ROOH is easy to further oxidize to RO•, which can seize H from the adjacent PUFAs, start a new round of lipid oxidation reaction, cause a large amount of oxidation of PUFAs, and damage the integrity of the biofilm, eventually leading to cell death (56).

### Regulation mode of ferroptosis

Ferroptosis is essentially the cell death caused by the imbalance of the production and clearance system of lipid ROS, which causes a large consumption of PUFAs on the biofilm. Therefore, the factors influencing the production or the clearance process of lipid ROS can regulate the ferroptosis process.

#### Inducible factors of ferroptosis

GPX4 is the main regulator of ferroptosis (54). The inhibition of GPX4 expression by RNAi can induce ferroptosis, while the overexpression of GPX4 can resist the RSL3-induced ferroptosis (54). RSL3 can covalently bind with selenocysteine at the active site of GPX4 and directly inhibit the enzyme activity of GPX4, thus inducing ferroptosis (54); as an inhibitor of GPX4, the artificial small molecule compound ML162 can also induce ferroptosis (57) (**Figure 2**).

As the elimination of lipid ROS by GPX4 requires the consumption of reduced GSH, the factors affecting the synthesis of GSH can affect ferroptosis. GSH is formed by one molecule of glutamic acid and one molecule of glycine under the catalysis of glutamate–cysteine ligase and glutathione synthetase (58). Erastin and its analogues can inhibit the Xc^-^ system, hinder the uptake of cystine, block the source of intracellular cysteine, and inhibit the synthesis of GSH (55). Buthionine-(S, R)-submaple imine (BSO) can inhibit glutamate–cysteine ligase, deplete glutathione, lead to the accumulation of lipid ROS, and induce ferroptosis (55) (**Figure 2**).

#### Inhibiting factors of ferroptosis

In the past, studies on ferroptosis mainly focused on GPX4, while the recently discovered ferroptosis regulatory pathway, which is mediated by ferroptosis suppressor protein 1 (FSP1) and independent of GSH peroxidase, suggests that ferroptosis may be regulated by multiple upstream mechanisms (**Figure 2**). FSP1 was originally named AIFM2 (apoptosis inducing factor mitochondrial 2) because it is homologous with AIFM1, but it lacks mitochondrial localization and has no function of promoting apoptosis (59). The N-terminal of FSP1 contains a myristoylation motif. After myristoylation, FSP1 is located in the plasma membrane through its N-terminal. The myristoylated FSP1 can play the role of its oxidoreductase to reduce CoQ10 and thereby reduce lipid peroxidation and the sensitivity of cells to ferroptosis (59, 60).

Since ferroptosis is caused by the excessive production of lipid ROS, the application of antioxidants such as ferrostatin-1, trolox, butylated hydroxytoluene, and tert butyl hydroxyanisole can eliminate lipid ROS, thereby inhibiting the process of ferroptosis. In addition, since the lipid oxidation process requires the participation of ferrous ions, the application of iron ion–chelating agents such as desferrilamine and cyclopidone can reduce the production of lipid ROS, thereby inhibiting ferroptosis (61) (**Figure 2**).

## Hippo pathway and ferroptosis

Recent studies have shown that cell density information can regulate the activity of YAP/WW domain containing transcription regulator 1, a transcription coactivator, through the Hippo pathway, thus regulating the sensitivity of cells to ferroptosis.

Wu et al. (62) showed that cell density information can affect the sensitivity of cells to ferroptosis through the E-cadherin-NF2(Merlin)-YAP-TEAD-ACSL4/TFRC (ACSL4, acyl-CoA synthetase long-chain family member 4; TFRC, transferrin receptor 1) pathway. TFRC and ACSL4 are important regulators of ferroptosis (63, 64). TFRC can cooperate with transferrin to transport iron ions from extracellular to intracellular (63). ACSL4 mediates the synthesis of polyunsaturated ω6 fatty acids arachidonic acid (AA) and docosahexaenoic acid (AdA) and the synthesis of arachidonic acid coenzyme A (AA CoA) and docosatetraenoic acid coenzyme A (AdA CoA). AA CoA and AdA CoA combine with cell membrane phospholipid molecules (phosphatidylinositol and phosphatidylethanolamine) and are important substrates for ferroptosis (64, 65).

For HepG2, PC9, H1650, HCT116, and other cell lines, with the increase of cell density, the sensitivity of cells to ferroptosis induced by erastin, RSL3, and the deficiency of cystine decreased. When the cell density increases, the adhesive connection between cells and E-cadherin at the junction increases. E-cadherin can transfer the cell density information to the Hippo pathway through NF2 (62). On the one hand, NF2 reduces the degradation of LATS1/2 by inhibiting E3 ubiquitin ligase CRL4-DCAF1 (66). On the other hand, NF2 mediates the phosphorylation of LATS1/2 *via* MST1/2 (25, 29), thereby enhancing LATS1/2 activity. Activated LATS1/2 phosphorylates YAP and promotes its cytoplasmic localization. At this time, the expression of ACSL4 and TFRC genes downstream of YAP-TEAD was inhibited. Due to the lack of iron ions and the substrate AA CoA and AdA CoA, the sensitivity of cells to ferroptosis decreased (62).

Different from the said cell lines, BT474 cells have a low sensitivity to ferroptosis whether at a higher or lower cell density, which may be due to the high expression of E-cadherin in BT474 cells even at a lower cell density. MDA-MB-231 cells are highly sensitive to ferroptosis even when the cell density is high; this is because the expression of E-cadherin in MDA-MB-231 cells remains at a very low level even when the cell density is high (62).

In addition, Yang et al. (67) found that in renal cell carcinoma, cell density information can regulate ferroptosis through the transcription coactivator TAZ (WW domain-containing transcription regulator protein 1, WWTR1). When the cell density is low, the activated TAZ can promote the expression of epithelial membrane protein 1 (EMP1) and then upregulate the level of NADPH oxidase 4 (NOX4). NOX4 increases the level of lipid ROS in cells and induces ferroptosis.

## Summary and prospects

YAP has been considered to be related to cell growth and proliferation. In most tumors, YAP is highly expressed, which can inhibit tumor cell death and increase its migration and invasion ability. In addition, because the high expression of YAP is related to the therapeutic resistance of tumors, the research and development of therapeutic methods targeting YAP and Hippo pathways have always attracted much attention. In a few tumors such as lymphoma and multiple myeloma, YAP promotes cell apoptosis and plays a role similar to that of a tumor suppressor. In different tumors, how the different effects of YAP on cell death are regulated, and whether they are related to the characteristics of tumor cells or different tumor microenvironments, need further discussion.

In addition, studies related to ferroptosis showed that under different cell density information, the sensitivity of cells to ferroptosis regulated by YAP was different: Under high cell density, the activity of YAP decreased, and the sensitivity of cells to ferroptosis decreased accordingly; at low cell density, YAP activity increased, and the sensitivity of cells to ferroptosis increased. Different tumor tissues have different cell densities. It may become a new idea for a specific tumor treatment to induce the ferroptosis of tumor cells with low cell density by regulating the Hippo pathway. However, there are still many problems with this idea, such as how to define human tumors with low cell density, how to specifically induce the ferroptosis of tumor cells without damaging normal tissues, and whether the induction of ferroptosis *in vivo* will lead to a large-scale imbalance between oxidation and antioxidation, thus causing irreversible damage to human normal tissues.

## Author contributions

JX and MJ analyzed and interpreted the data. XD collected information. JX and MJ worked equally. All authors contributed to the article and approved the submitted version.
